# Hospitals in India

**Published:** 1894-07-07

**Authors:** 


### HOSPITALS IN INDIA.

### II.—THE MADRAS PRESIDENCY.

### 1.—State Hospitals in the Presidency Town and at Mofussil Stations.

In accordance with instructions from Government the report on the working of the State hospitals in the Madras Presidency is furnished separately from that detailing the work of the civil hospitals and dispensaries, and this note, therefore, deals only with those institutions which come under the category of Class I., maintained wholly by the Government. The report under review is the full triennial one, and consequently the figures for 1891 are compared with those for 1888. The following statement compares the working and cost of the institutions in these two years, and shows how great an increase there has been during the past three years in the medical relief afforded to the people of the Presidency:—

| | Number of Hospitals. | Number of In-patients. | Number of Out-patients. | Daily Average of In-patients. | Mortality Percentage. | Number of Operations. Major. | Number of Operations. Minor. | Amount recovered from paying patients. | Total expenditure, including amount in preceding column. |
|---|---|---|---|---|---|---|---|---|---|
| | | | | | | | | Rs. | Rs. |
| 1888 | 14 | 11,951 | 81,927 | 529·49 | 7·91 | 1,885 | 3,456 | 10,239 | 2,73,019 |
| 1891 | 18 | 14,487 | 101,356 | 838·28 | 5·86 | 3,286 | 8,657 | 11,886 | 3,10,767 |

Practically all the available information is contained in the above table, and there is little to add. Being State hospitals the expenses connected with the working of these institutions were wholly borne by Government. In addition to the sum recovered from paying patients, Rs.197 were realised from various sources; the rest came from Government. In the expenditure statistics salaries represent Rs.95,094 1a. 3p., and European and bazaar medicines Rs.32,250 10a. Diets cost Rs.91,229 8a. 7p., or on an average 4a. 9p. each. The difference was spent on buildings and miscellaneous charges. Appended to the report are certain professional papers from the special hospitals in the Presidency town, which are of considerable interest from a professional point of view, and testify to an enormous amount of work done unostentatiously in these valuable institutions.

